# Experiences of mpox illness and case management among cis and trans gay, bisexual and other men who have sex with men in England: a qualitative study

**DOI:** 10.1016/j.eclinm.2024.102522

**Published:** 2024-03-12

**Authors:** T Charles Witzel, Andrew Ghobrial, Romain Palich, Hannah Charles, Alison J. Rodger, Caroline Sabin, Alex Sparrowhawk, Erica R.M. Pool, Mateo Prochazka, Roberto Vivancos, Katy Sinka, Kate Folkard, Fiona M. Burns, John Saunders

**Affiliations:** a Institute for Global Health, University College London, London, UK; b AP-HP.Sorbonne Université, Paris, France; c UK Health Security Agency (UKHSA), London, UK; d National Institute for Health and Care Research (NIHR) Health Protection Research Unit (HPRU) in Blood Borne and Sexually Transmitted Infections at UCL in Partnership with (UKHSA), London, UK; e Terrence Higgins Trust, London, UK; f World Health Organization, Geneva, Switzerland; g NIHR HPRU in Emerging and Zoonotic Infections at the University of Liverpool, Liverpool, UK; h NIHR HPRU in Gastrointestinal Infections at the University of Liverpool, Liverpool, UK

**Keywords:** Gay, Bisexual and other men who have sex with men, Transgender people, Mpox, Qualitative research, (re)emerging infectious diseases, Sexual health

## Abstract

**Background:**

The 2022–2024 global mpox outbreak, occurring primarily in the sexual networks of gay, bisexual and other men who have sex with men (GBMSM), has not been accompanied by a focus on patient perspectives of illness. We explore the experiences of GBMSM diagnosed with mpox in England to understand needs for social and clinical support.

**Methods:**

In-depth interviews (March/July 2023) were conducted with 22 GBMSM diagnosed with mpox in 2022, randomly selected from a national mpox surveillance database, and 4 stakeholders from clinical/community-based organisations. Interviews covered experiences of illness, testing, diagnosis, treatment and contact tracing, and were recorded, transcribed and analysed with a thematic framework.

**Findings:**

Media coverage drawing on homophobic stereotypes around sex between men contributed to feelings of stigma and shame. GBMSM living with HIV appeared to cope better with mpox stigma, drawing on their experiences of being diagnosed with HIV for resilience. Younger GBMSM with less experience of stigmatising illness found mpox diagnosis more traumatic and sometimes required support beyond what was provided. Accessing testing could be complicated when healthcare professionals did not recognise mpox symptoms. Men felt information on course of illness, isolation and vaccination after recovery was often inconsistent and contradictory. GBMSM described that care from sexual health and infectious disease units usually better met their emotional and medical needs. This was frequently linked by men to these services having skills in working with the GBMSM community and managing infection risk sensitively. General hospital services and centralised contact tracing could increase feelings and experiences of stigma as some staff were perceived to lack skills in supporting GBMSM and, sometimes, clinical knowledge. Long-term impacts described by men included mental health challenges, urethral/rectal symptoms and life-changing disability.

**Interpretation:**

In this study stigma was a central feature of mpox illness among GBMSM and could be exacerbated or lessened depending on the clinical and social support provided. Involving communities affected by outbreaks in co-producing, planning and delivering care (including contact-tracing) may help improve support provided.

**Funding:**

TCW, AJR, AS and FMB received support from the 10.13039/501100000272National Institute for Health and Care Research (NIHR) under its Programme Grants for Applied Research Programme (Ref: NIHR202038). CS and JS receive support from the National Institute for Health and Care Research Health Protection Research Unit (NIHR HPRU) in Blood Borne and Sexually Transmitted Infections at UCL in partnership with UKHSA; RV receives support from the NIHR HPRU in Emerging and Zoonotic Infections and NIHR HPRU in Gastrointestinal Infections. The views expressed are those of the author(s) and not necessarily those of the 10.13039/501100000272NIHR, UK Health Security Agency, 10.13039/100004423World Health Organization or the 10.13039/501100000276Department of Health and Social Care.

**Research in context**

**Evidence before this study**

With the aim of identifying research on the lived experiences of GBMSM diagnosed with mpox, we searched PubMed from inception until November 2nd 2023 using the following terms: [Sexual and Gender Minorities OR Homosexuality OR homosexuality male OR Gay OR Bisexual OR LGBT] AND [MPOX or MPXV OR monkeypox virus]. This yielded 327 results, none of which provided information on patient experience beyond clinical data; through our networks we identified two publications and one conference abstract of relevance.

Two studies from the UK, including 12 GBMSM previously diagnosed with mpox in total, focused on mpox prevention/testing measures and the utility of social media in health promotion. Research with 13 GBMSM in Australia explored their lived experience of mpox and found illness and isolation was extremely distressing, that many were dissatisfied with the care they received and that some experienced stigmatising attitudes from healthcare professionals who lacked skills in supporting GBMSM.

**Added value of this study**

This study demonstrates that GBMSM contextualised mpox severity in relation to HIV and other sexually transmitted infections and reported a wide range of experiences of stigma, ranging from internalised felt stigma to active discrimination in healthcare settings, in workplaces and online. Younger, HIV negative GBMSM coped less well with mpox stigma, whereas older men who were living with HIV drew on resilience from past experience of HIV diagnosis. Comprehensive information on illness and clinically effective, culturally competent care may reduce stigma for GBMSM experiencing mpox. We also explore the prolonged, negative impact of mpox on the mental health of some GBMSM with pre-existing mental health challenges.

**Implications of all the available evidence**

Stigma is a central feature of mpox illness experience for GBMSM. Clinically appropriate and culturally competent care may help to reduce stigma for those impacted by (re)emerging infectious diseases. Involving affected communities in co-producing approaches to contact tracing and clinical care may improve responses and should be matched by appropriate funding.

## Introduction

In May 2022, a rapidly expanding global outbreak of the Clade IIb lineage of mpox (formally monkeypox) was identified among the sexual networks of gay, bisexual and other men who have sex with men (GBMSM), first in the UK, Portugal and Spain, before spreading across Europe and the Americas.^1^^,^^2^ Mpox cases declined in Europe and North America from August 2022, aided by community mobilised public health education and behaviour change, as well as vaccination.^3^^,^^4^ Ongoing transmission has remained an issue in other areas, especially Latin America, Africa and Asia.^5^^,^^6^ This outbreak, and the unique clinical presentation among GBMSM suggest sexual and other very close skin contact has had a role in sustaining transmission.^7^, ^8^, ^9^ Concerns exist that mpox outbreaks will continue among GBMSM globally due to low vaccination rates in many countries.

In the UK and other settings, mpox put extreme pressure on sexual health services (SHS). As the primary providers for mpox testing and, latterly, vaccination, SHS rapidly developed and implemented mpox infection control measures, because of the initial status of mpox as a high consequence infectious disease (HCID), while managing increased workloads. This occurred against a backdrop of long-term underfunding which,^10^^,^^11^ alongside disruption from the COVID-19 pandemic, contributed to SHS capacity issues.

Clinical care evolved during the outbreak and has included pain management, hydration, antibiotic treatment of secondary infections, post-exposure vaccination for contacts with the smallpox vaccine (MVA-BN), and compassionate use of antivirals (Tecovirimat [TPOXX]).^9^^,^^12^

Due to the overtly visual manifestation, stigma is likely to be a central feature of the experience of mpox illness.^13^^,^^14^ This may manifest as experiences of social judgement and discrimination (enacted stigma), or as internalised shame and fear of encountering enacted stigma (felt stigma).^15^ Stigma from highly visible skin lesions and potential scarring may intersect with stigmas associated with sex between men, sex itself, and homophobic narratives around groups with increased mpox risk (e.g. GBMSM with frequent partner exchange).^14^^,^^16^, ^17^, ^18^

Qualitative research surrounding mpox has focused on acceptability of public health measures amongst those at risk, including vaccination and contact tracing.^19^^,^^20^ There are limited data on patient experiences of the care pathway, including testing, treatment, and long-term impacts of mpox. Research is required to understand the experiences of GBMSM diagnosed during the 2022–2024 outbreak to inform care which improves health and well-being outcomes and addresses stigma.

We **aim** to explore the experiences of GBMSM (cis and transgender) diagnosed with mpox in England and understand their needs for social and clinical support. We do this by exploring the social and emotional dimensions of mpox and identifying clinical and social support needs, including impacts on mental health.

## Methods

### Ethics statement

Ethical approval was provided by the UK Health Security Agency's (UKHSA) Research Ethics and Governance Group [ref: 522]. Participants provided electronically recorded consent.

### Study design and setting

We conducted in-depth interviews with GBMSM diagnosed with mpox between March and July 2023. We also conducted interviews with stakeholders involved in the response to provide nuance and triangulate findings.

Interviews were conducted by the first and second author, and analysis by the first and third, all of whom are gay cisgender men. Patient and public participation input was provided by the seventh author.

### Sampling and recruitment

Eligible men (cis and transgender) were aged 18 years or older, reported sex with men and had a diagnosis of mpox in 2022. GBMSM were recruited from the UKHSA surveillance database of all individuals with confirmed mpox diagnoses in England. Text messages containing a link to a registration survey with study information, screening and demographic questions (supplementary 1) were sent to a random selection of individuals on the database. Stakeholders were recruited from the networks of investigators based on sector (e.g. clinical, community) and role in the outbreak.

Purposive quota sampling ensured inclusion of GBMSM who had been hospitalised and/or non-UK born. Participants were given £30.

### Data collection

Topic guides (supplementary 2) were developed by exploring existing literature and discussing emerging priorities amongst the study team.

GBMSM interviews covered experiences of mpox, navigating care, clinical services and contact tracing. Interviews finished with demographic and behavioural questions. This topic guide was piloted with two interviewees then refined for clarity and flow.

Stakeholder interviews covered outbreak preparedness, the needs of people diagnosed with mpox, and stigma. This was piloted with one stakeholder and did not require refinements.

Interviews were conducted online (Microsoft Teams/Zoom), audio-recorded and transcribed verbatim. The audio quality for one interview was too poor to be transcribed; the interviewer wrote structured notes and quotations from this recording.

### Statistical analysis

We tabulated study recruitment data and baseline sample demographics using Microsoft Excel.

### Qualitative analysis

Our qualitative analysis fused thematic and framework approaches.^21^^,^^22^ The first, second and third authors read overlapping selections of transcripts and developed themes, then compared and reformed these. A team-wide meeting reviewed and refined themes which were then reorganised into a hierarchical framework. The first author piloted the framework on two transcripts and made adjustments. The final framework (supplementary 3) was applied to all study transcripts.

We developed a typology of mpox illness severity to explore findings and compare experiences between groups qualitatively. This typology (Table 1) combined clinical and self-assessed indicators and classified participants as having mild, moderate or severe illness, drawing on previous work.^23^ Alongside this comparative analysis, negative case analysis was used to identify and explore divergent perspectives.^24^ Stakeholder interviews were used to further triangulate perspectives, adding depth and nuance. We used QSR NVivo 14 for data management and organisation.

**Table 1:** Mpox severity definitions used in qualitative analysis.

Severity	Indicators
Mild	• 1–10 lesions • No or brief fever • Only visited healthcare services for testing/diagnosis
Moderate	• 10 or more lesions and/or very severe anorectal pain • High fever >1 day • Multiple visits to healthcare services as outpatient for treatment/pain management
Severe	• Stayed in hospital overnight or longer because of mpox illness

### Role of funding source

The funders played no role in study design, data collection, analysis, interpretation or writing the manuscript. The first, second and third authors had access to all study data. All authors agreed to submit for publication.

## Results

We sent out 522 invitations to a random selection of individuals diagnosed with mpox from a total database of 3,581, leading to 60 study registrations, of whom 22 were interviewed. See Table 2 for sample demographics. We also interviewed 4 stakeholders involved in the response, 2 each from clinical and community organisations. Stakeholders worked in both leadership and service delivery roles.

**Table 2:** Demographic details of all GBMSM interview participants.

Demographic detail	N (%)
**Age**	
18–25	2 (9.1)
26–35	7 (31.8)
36–45	6 (27.3)
46+	7 (31.8)
**Sexual Orientation**	
Gay	18 (81.8)
Bisexual	4 (18.2)
**Gender**	
Cisgender	21 (95.5)
Transgender	1 (4.5)
**UK born**	
Yes	14 (63.6)
No	8 (36.4)
**Ethnicity**	
White	18 (81.8)
Asian^a^	1 (4.5)
Black^a^	1 (4.5)
Latin American	2 (9.2)
**HIV status**	
Positive	6 (27.3)
Negative	5 (22.7)
Negative, taking HIV pre-exposure prophylaxis	11 (50.0)
**Diagnosis**	
May–June 2022	7 (31.8)
July–August 2022	13 (59.1)
September 2022 onwards	2 (9.1)
**Severity**	
Mild	10 (45.5)
Moderate	7 (31.8)
Severe	5 (22.7)

a Including mixed ethnicities.

Analysis focused on two meta-themes: 1) social and emotional impacts of mpox and 2) experiences of healthcare provision and recovery.

### Social and emotional impacts of mpox

This meta-theme examines emotional reactions to diagnosis and how men contextualised mpox in relation to other sexually transmitted infections (STIs). Following, we explore the multifaceted experiences of mpox stigma and the impact of mpox on sex and relationships.

#### Emotional reactions: contextualisation and fears

The emotional reactions of GBMSM diagnosed with mpox varied considerably. GBMSM with mild illness typically expressed shock and surprise upon testing positive; often reflecting they did not feel at risk. This was consistent throughout the outbreak. In contrast, GBMSM with moderate and severe mpox were usually less shocked and more accepting of diagnosis as they had more typical presentations and, in some cases, were already profoundly unwell and admitted to hospital.

GBMSM often contextualised mpox in relation to HIV and other STIs. Because there was no generally accessible antiviral treatment for mpox and because of the isolation requirements, it was felt to be more severe than bacterial STIs such as chlamydia, gonorrhoea and syphilis. However, as mpox is self-limiting and not usually life-threatening, it was felt to be less severe than HIV:

*In terms of the process and the feelings of actually finding out, it felt more similar to when I was diagnosed with HIV. In that there was that kind of denial. And is it that, is it something else, what could it be.* […] *And obviously the consequences are different because the consequence of this was isolation for three weeks. Whereas the consequences of catching HIV that, you know, you have to live with it for the rest of your life.*

(Cisgender White gay man, aged 36–45, moderate illness)

For a small minority, mpox was seen as less serious than syphilis because syphilis antibodies used in routine testing are detectable beyond recovery and later tests are therefore harder to interpret. One participant who expressed relief when diagnosed with mpox explained: *Because I knew that it would pass, but it didn't have any-it doesn't affect your* [routine] *testing afterwards does it? I mean it's annoying but it's not drastic.* (Cisgender, mixed White and Asian gay man, 26–35, mild illness)

Across all severity groups, GBMSM reported similar fears at diagnosis. The primary concern for many was how the illness would progress, if they would be left with scarring and what complications might arise. This was compounded by a lack of comprehensive information about the course of mpox illness, especially as it related to the 2022–2024 outbreak rather than previous clinical experiences from Central/West Africa. This issue appeared to be most pronounced early in the outbreak, but present throughout. According to a man diagnosed in May/June:

[…] *there was a lot of doom scrolling definitely, trying to look up online everything about this illness, because there wasn’t a lot of information out there, about what happens next, what’s going on? Is it going to spread? Are they going to be elsewhere?*

(Cisgender White gay man, aged 26–35, moderate illness)

Those most concerned about longer-term impacts were typically men who had been hospitalised and experienced more serious sequalae, such as neurological events. This concern was compounded due to a lack of information provided by clinical teams, again related to limited understandings of outcomes early in the outbreak. For these men, concerns about longer-term impacts lingered far beyond recovery.

#### Intersecting multiple stigmas

Stigma was a central feature of mpox illness. For many, mpox was linked with homophobic narratives including promiscuity, illness and disease. These were felt across all severity groups and often linked to sensationalised media representations, but also to public health information disseminated through health bodies. The use of stigmatised gay identities by the media especially served to obscure the broader relevance of the mpox outbreak as some men did not recognise themselves within these narratives. A transgender participant felt that because messaging highlighted cisgender gay men, it didn't feel relevant to him:

*I think, I mean the impacts of monkeypox as well, there is a social idea of it so a lot of people assume it’s obviously just gay men, all of that sort of stereotypes around it. When I was coming out being, “*Oh yes, I’ve got monkeypox*.” A lot of people went straight to the stereotype of assuming I’m going around sleeping with a lot of guys etc.*

(Transgender White bisexual man, aged 18–25, mild illness)

Felt stigma was a key experience for many. GBMSM who were older, had mild/moderate illness and who were living with HIV coped better with mpox felt stigma, and often linked this with prior experience of HIV as a stigmatising illness. In contrast, younger GBMSM who were HIV negative and did not have the same experience to draw upon appeared to cope less well with mpox related stigma, finding it a traumatic and personally threatening experience.

*It felt like I was- the best way to describe is probably dirty. I felt actually really not self-conscious because I knew that nobody else would see them* [mpox lesions] *other than obviously the doctors and nurses. But I felt like I was judging myself basically for having them. I can remember sitting at home and I was just crying because I was like what do I do about these?*

(Cisgender White gay man, aged 26–35, mild illness)

While felt stigma was very common, enacted stigma, was less so, likely because many successfully navigated disclosures. This was especially the case when men correctly identified supportive individuals in their lives, or conversely, when they attributed their isolation to COVID-19 illness rather than mpox.

Enacted stigma occurred within the health service, from sexual contacts, on social media, and in workplaces. Many were subtle or one-off comments, while others were more serious and included periods of abuse. One participant diagnosed early in the outbreak who shared their diagnosis on social media experienced intense trolling online:

[…] *I was receiving a lot of homophobic abuse. Not just from straight people, but I was getting it from the LGBT community as well. So they were calling me disgusting, had I learnt nothing from COVID? Likening monkeypox to the AIDS crisis and all that kind of stuff*.

(Cisgender White gay man, aged 36–45, mild illness)

Three men experienced inappropriate, unsanctioned disclosures of their mpox diagnosis by managers to other colleagues. Two of the men were not open about their sexual orientation at work and this resulted in upsetting periods of gossip and ridicule which were difficult to manage.

Men experiencing felt and enacted stigma linked these to media representations of mpox, and some identified this stigma as the most significant they had experienced because of broader improvements in the rights of GBMSM:

*After I left the clinic, I got very emotional. Not because I had monkeypox, I didn’t feel like I did anything wrong* […] *But I just felt like- I felt let down by the way that, the discourse, and the way that the infection, the virus or whatever it is was being portrayed as well. Yes, it just took me to a place where I just didn’t expect to feel in terms of my experience as you know, a gay man, with lots of privilege in lots of ways. But, usually I felt like I had dignity in the* [health] *service and the way I am treated by the government and the likes of that. And it just kind of really sped away suddenly*.

(Cisgender White gay man, aged 26–35, moderate illness)

When describing the societal impacts of mpox stigma, some men and all stakeholders attributed perceived government inaction on mpox as due to the links of the pathogen to stigmatised sexual minorities. These structural failings were often compared to the early reactions to the AIDS crisis.

#### Mpox, sex and relationships

All GBMSM interviewed understood the necessity to avoid sexual contact while recovering. For those with moderate/severe disease, this was relatively uncomplicated as symptoms meant sex was profoundly undesirable. For some with mild illness and lingering disbelief in their diagnosis, abstinence was more challenging. A man who had a single, small mpox lesion on his leg described: *Because I didn't have signs telling me that I had something to stop me, I still had the want to go out and do what I do.* (Cisgender White gay man, aged 26–35, mild illness).

Longer-term impacts on feelings about sex were varied. For many with very mild and transient illness, mpox had no impact on their feelings about sex in the future. For others, including those with more serious and/or painful illness, mpox led to reductions in sexual partner numbers. This was sometimes transient and linked to mpox being a frightening experience; for others having mpox was associated with feelings of loss of control of behaviour leading to a wider, longer-term revaluation.

*Definitely you change a bit, you know, because I mean we are in an open relationship, so you go wild sometimes, you know what I mean? And today I can see myself a bit more reserved. I’m not going to lie and say, oh I’m not going to parties, I don’t do this. I am still doing, but with a step back*.

(Cisgender Latin American gay man, aged 36–45, moderate illness)

Most men were not in long-term relationships; for those who were, relationships were primarily non-monogamous. Although men described various negative relationship impacts of mpox, such as reduced sexual contact and discomfort re-initiating sexual behaviour following recovery, these were transient and did not lead to relationship breakdowns.

### Experiences of healthcare provision and recovery

This second meta-theme focuses on the experiences of GBMSM navigating mpox clinical care pathways, including tensions between belief/disbelief of men with mpox and their healthcare providers during testing and diagnosis, as well as the importance of clear, comprehensive information on illness for those experiencing mpox. We also explore experiences of care during clinical management and contact-tracing and needs around longer-term support.

#### Managing (dis)belief: initial illness and testing

For many the long incubation period and number of contacts made transmission events difficult to ascertain. Although sexual activity was the presumed route of most acquisitions, 3 identified non-sexual contacts as most likely. These men were sometimes not believed in health services, leading to feelings of frustration and mistrust.

*Every professional I come across doesn’t seem to believe me and I would be very frank with people in saying this is how I got any sort of STI infection. So at the time he* [a friend] *had just come back from* [redacted]*. We met at a bar and I was sat next to him and my partner was facing him. He shared his phone with me and he was showing me pictures* […] *that was the mechanism that I can only think of*.

(Cisgender White gay man, aged 26–35, moderate illness)

Initial signs of mpox illness were diverse. GBMSM with mild illness had typically identified one or more blister-like lesions appearing on the anus, genitals or in the mouth/throat. These were often initially mistaken by men for pimples or cold sores. This was a consistent issue throughout the outbreak. For men with moderate and severe illness, usually several lesions developed simultaneously, although for some (n = 2) flu-like symptoms were the first indication.

Most men with moderate/severe illness were diagnosed promptly due to the distinctive nature and severity of their symptoms. A minority (n = 5) who sought testing, particularly those with mild illness, described issues with misdiagnoses and diagnostic delays, especially earlier in the outbreak when healthcare staff were less familiar with mpox symptoms. During these testing delays, symptoms were attributed by staff to herpes (n = 1), syphilis (n = 2) or other conditions (n = 2) and testing was refused or mpox was not considered. One participant, who spent several days as an inpatient because of symptoms which were mistaken for a flare of a pre-existing chronic illness, described his experience of convincing those managing his care to test him for mpox:

*And I think it was around this time, because I had this spot here, my friend had had monkeypox, and I’m gay. And so, I just started thinking, ‘*Oh, could it be monkeypox?*’ And so, I think I may have suggested this to one of the surgeons, who I think kind of dismissed it and didn’t really think much about it.* […] *The* [redacted specialist] *came and saw me* […] *I sort of showed him this lesion on my hand and said I wondered whether if it could be monkeypox. And I think he kind of took that idea more seriously*.

(Cisgender White gay man, aged 36–45, severe illness)

Following testing, men who had more lesions and/or more severe disease generally had an easier time believing that they had mpox, again because their symptoms were distinctive. Men who had very mild illness, especially those diagnosed because healthcare workers recognised lesions that the men had attributed to other causes (n = 2), had a more difficult time and in one case expressed significant ambivalence as to whether the diagnosis was correct.

#### Information quality and inconsistency

During testing and diagnosis most men received information from healthcare providers performing testing. This was usually sensitively delivered, met men's needs and could help lessen stigma:

[…] *I was asking how many cases have they seen? Was it frequent? What’s the recovery time? Is it normal to feel the way I’m feeling in terms of sickness?* […] *I did talk to them about the shame stigma element of it. I was like ‘Have you found that it is being more talked about as a gay and bisexual disease?’ and they said yes they had. But they also did say, ‘we know that’s not a true reflection.’ So they were really good in that sense where they did say, ‘it’s out there, it’s what the media is reporting but it’s just an infection that anyone can have.’*

(Cisgender White gay man, aged 26–35, mild illness)

In contrast, accessing comprehensive information from trusted sources after diagnosis was often challenging for men with mild illness. While many looked online, others attempted to recontact either clinical or contact-tracing services. This experience was often described as fraught because contact tracing especially was perceived as inaccessible and challenging to access directly, leading to multiple unsuccessful attempts for some. While many reported positive experiences of UKHSA contact-tracing generally, some personnel were felt by GBMSM to lack clinical knowledge and/or efficacy, especially around accessing vaccination for contacts.

Although self-isolation was acceptable, the national guidelines were perceived to be inconsistent, unclear and frequently changing early in the outbreak. Stakeholders highlighted that this was because of the rapidly evolving nature of the evidence base and potentially sub-optimal public health communication. Among men, there was some confusion about how long self-isolation lasted, although many correctly identified scabs falling off with healed skin underneath as the cut-off.

One man, diagnosed July/August, who was being cared for as part of a virtual ward (a remote clinical service for those with moderate mpox not requiring in-patient treatment) described how healthcare staff threatened to report him to the police for ending 6-weeks of self-isolation before they judged him fit to do so. This was despite him self-assessing that he met criteria as all his scabs had fallen off with fresh skin underneath.

*Yes, it did get to the point where I said to the hospital at the end of the isolation, I refused point blank to speak to them because they were saying*, ‘We're going to contact the local police*.’ And I was like*, ‘Well do what you need to do, I'm refusing to speak to you, please transfer my care back to the clinic.’

(Cisgender White gay man, 26–35, moderate illness).

Finally, following mpox recovery, information on vaccination was inconsistent. Few were proactively offered vaccination and several who sought it (n = 4) were denied first or second doses because they were presumed immune. This issue persisted throughout our data collection period (March/July 2023), and led to feelings of vulnerability to reinfection, especially given emerging case reports from the UK and elsewhere.

#### Cultural competence and clinical care

The ability to engage with healthcare and contact tracing providers in an environment and manner free from stigma was very important for men diagnosed with mpox. For this reason, many preferred to seek care at sexual health clinics, which were felt to have the cultural skills necessary to support GBMSM without worsening stigma. Although UKHSA contact-tracing was generally seen to be well managed, some men (n = 4) identified a lack of skills with working with the LGBTQ+ community. This was described most commonly by those who were ill during the early stages of the outbreak, when mpox was still classed as a HCID (pre-July 2022) and contact-tracing was more rigorous. Because of this, men sometimes felt interventions were overly intrusive, and exacerbated stigma:

**Participant**: *I don’t know what the team is like at UKHSA, but even having, I don’t know, an LGBT person call you, because for example, this interview now, speaking to you, it’s a lot more comfortable than it would be if I was speaking to a straight guy. Because there’s kind of like-*

**Interviewer**: *a shared understanding.*

**Participant**: *a shared understanding, exactly, and I think that would have been very helpful because it felt very kind of, almost like an interrogation, which makes you feel like you've done something wrong, and I was like, I've only been on holiday*.

(Cisgender White gay man, aged 36–45, mild illness).

Many men described that good mpox clinical care meant having adequate follow-up which was responsive to their needs. Generally, despite struggling under intense workloads, SHS were felt to perform well in meeting the physical health and psychosocial needs of GBMSM with mild illness throughout the outbreak. Some individuals were provided with well-being checks online or via telephone and offers of practical support to isolate. Others described feeling adrift with little to no contact despite experiencing profoundly unpleasant symptoms and requiring additional support. This primarily impacted those unable to disclose their mpox to their social networks.

GBMSM described that service providers’ clinical knowledge and skills around managing transmission risks sensitively was vital and could help lessen felt stigma. Conversely, men linked experiences of enacted stigma to the lack of such skills among non-specialist clinical staff. All men who had contact with general hospital services (such as Accident and Emergency) after receiving an mpox diagnosis (n = 4) reported some degree of enacted stigma from staff, usually related to infection control measures. This ranged from mild hostility to being instructed to wait outside the hospital supervised by security staff:

*They told reception that security had to protect, to cover me and not let me move. I had to sit on one seat outside in the open with two security standing around me* […] *I looked like a patient under mental health 136* [section] *mate, that’s what it looked like*.

(Cisgender White gay man, aged 46+, severe illness)

In contrast, some men described that larger hospitals with specialist infectious disease units were better equipped to deal with patients experiencing moderate and severe mpox illness. This was attributed by stakeholders to having staff onsite versed in managing infectious diseases, including HCIDs.

For many, good clinical care revolved around having adequate symptom control. Although practices evolved during the UK outbreak, pain management was a persistent issue. Most men with mild/moderate mpox requiring it were provided with some form of pain management. This met their needs to a varying degree. However, 5 were denied any medication beyond paracetamol and ibuprofen. This was usually earlier in the outbreak but also occurred when men were treated in sexual health clinics that had seen fewer cases. According to man diagnosed late in the outbreak:

*They weren’t, bless them, they were not helpful at all* […] *my friend went to a clinic* [in London] *and because they had cases before* […] *they gave him medications and whatnot. My clinic didn’t give me any medication. They were like just isolate and you should get better. So I know we shouldn’t do it but* […] *he shared his medications with me. He was already getting better* […] *and I didn’t have any.*

(Cisgender mixed Black and White bisexual man, aged 18–25, moderate illness)

Two men hospitalised in infectious disease units were provided with excellent pain control and felt their needs were well met. Conversely, a further man diagnosed early in the outbreak had substantial issues with denial of pain management, leading to feelings of not being trusted by doctors, exacerbating stigma.

Another man with critical mpox illness felt that the care he received did not take adequate account of the severity of his condition and that this contributed to a catastrophic outcome. He had a prior HIV diagnosis, but was not engaged in care or on antiretroviral treatment and had a very low CD4 count. He was initially hospitalised briefly for mpox illness, discharged, readmitted for some time, then discharged again. He was subsequently readmitted with critical illness featuring mpox related tissue necrosis. He underwent extensive surgical debridement which led to life-changing disability. He remained in hospital for several months. This man felt that had staff recognised the severity of his condition and not discharged him that he would not have experienced such severe and life-changing complications.

*I felt their* [early admission] *focus was*, *I might be able to eat. They need to make sure, for me to be discharged* […] *that I was able to feed myself and that was their main focus. Nothing was ever really said about monkeypox, nothing was ever said. Potentially, if I had been identified, I* [would not have had this outcome].

(Cisgender White gay man, aged 46+, severe illness)

#### Seeking longer-term support

The majority with mild mpox experienced no longer-term impacts. For some, mild genital scarring caused recurrent felt stigma, especially with new sexual partners, providing an unwelcome reminder of a difficult period in their lives.

For men with moderate/severe illness, longer-term impacts were more common and ranged from tiredness (n = 3) to life-changing and permanent disability (n = 1). Three men described ongoing urinary and bowel issues requiring specialist management and repeated visits to clinics. For the man with life threatening tissue necrosis, the disabilities resulting from surgery have had serious impacts. He has ongoing difficulty in accessing the psychosocial/health services necessary to address his disability and mental health challenges as these services are often not equipped or flexible enough to meet the needs of individuals with complex trauma and depression.

Regardless of mpox severity, men with pre-existing mental health concerns (n = 5) described a deterioration in well-being, often substantial, linked with mpox. While most men associated this with stress and mpox stigma, others felt that there was a biological process underpinning this as the depression experienced was more profound and long-lasting than prior periods.

*I still get the drops. I mean I suffer from major depressive disorder as it is anyway, so I think that combined with that illness that does affect your mental health, it just made it a thousand times worse. It impacted me, rather than it being up, down, up down, it was just a long-term stream of depression* […] *I think there was obviously some long-terms impacts of it on the brain.*

(Transgender White bisexual man, aged 18–25, mild illness).

These men described struggling to access support and were left with unanswered questions about how long their symptoms might endure and potential biological mechanisms, questions echoed by stakeholders who identified evidence gaps in this area.

## Discussion

Since the outset of the 2022–2024 outbreak, case series have been critical in rapidly disseminating information about mpox.^9^^,^^25^^,^^26^ This has not been accompanied by a similar focus on the lived experience of those with the illness. This study focusing on the narratives of 22 GBMSM diagnosed with mpox, with additional depth drawn from 4 stakeholder interviews, is among the first exploring the needs of individuals with mpox illness qualitatively.

Emotional reactions to diagnosis varied; men with mild illness were often shocked as the diagnosis was unexpected, whereas positive results were anticipated by those with moderate and severe illness. Fears related to the course of mpox were compounded by a lack of comprehensive, detailed information from both clinical and national public health services.

Mpox was associated with substantial felt stigma, especially homophobic narratives linking promiscuity with illness and disease. This was by media coverage, and sometimes related to information provided by national health agencies. Internalised felt stigma was experienced by nearly all men in our sample. An unexpected finding, given sectoral discussions around emotional vulnerability during the outbreak, was that men living with HIV typically coped better with felt stigma than younger, HIV negative men. Some experienced enacted stigma in healthcare settings, from sexual contacts, in online spaces, and because of inappropriate disclosures by employers.

High-quality clinical care and psychosocial support is the cornerstone of effective mpox case management. While many had good experiences, some GBMSM described how a lack of clinical knowledge and cultural skills in supporting LGBTQ + communities could increase stigma. This was felt by men to be more common in services which did not routinely provide healthcare to GBMSM and in services which had less experience in managing transmission risks sensitively.

The social and emotional dimensions of mpox illness are complex. Beyond a recent study conducted in Australia which included smaller numbers of men with a narrower range of clinical presentations,^27^ there is a dearth of evidence in which to situate our research. More social science inquiry is required to understand the experiences of mpox illness in a range of settings, including low- and middle-income countries where the outbreak is ongoing.

Our exploration of the emotional responses to mpox are novel and illustrate how individuals contextualised their experiences in relation to HIV and bacterial STIs. We extend understandings of how homophobic tropes surrounding mpox, disseminated through the media, reinforced stigmatising views of GBMSM and contributed to both felt and enacted stigma. UK research conducted during the 2022 outbreak, primarily with those at risk of mpox, found that stigma could also act as a barrier to testing and, potentially to engagement with contact tracing.^19^ Indeed, the pervasiveness of stigma in our participant's narratives and its potential role in constraining health-seeking behaviour and leading to distress highlights the need for stigma reduction interventions during outbreaks of (re)emerging infectious diseases.

Clinical services had variable success in meeting the needs of individuals diagnosed with mpox. This included some missed opportunities for diagnosis, and inconsistent provision of pain management across services, the latter of which was unexpected. In addition, the man experiencing the most severe illness in our study felt his especially poor outcome was due to missed opportunities to manage his mpox effectively. These issues may be partly due to mpox previously being primarily limited to Central and West Africa and receiving little attention outside these areas.^28^ Wherever possible, during outbreaks, testing thresholds should be low to increase case finding. This may require scale-up of testing capability and training on identifying diverse signs of illness. Further research is required to understand clinician barriers and facilitators to providing adequate pain relief for this condition and to GBMSM.

A lack of cultural skills in meeting the needs of GBMSM in some services led to stigma, and sometimes, distress. This highlights the role of involving members of communities affected by outbreaks in co-producing, managing and delivering support, including contact tracing where stigma is likely to be a key intervention barrier. This should be done continuously from the outset of outbreaks and be accompanied by appropriate funding, especially for community-based organisations who often face financial precarity. This finding also underlines the importance of utilising services best equipped to meet the needs of populations affected by (re)emerging infectious diseases. For GBMSM experiencing mild illness this is likely SHS, and specialist infectious disease units for those more seriously unwell. Increased workloads related to outbreaks should also be accompanied with appropriate funding to reduce disruption to routine provision, a critical issue during the outbreak.^11^^,^^29^

Finally, several men reported that mpox worsened their mental health. While existing qualitative research explores the impact of the mpox outbreak on GBMSM generally,^30^ the mental health impacts of mpox itself, including potential contributing biological mechanisms, are poorly understood. This emerging area of focus remains an important research priority for future enquiry.^31^^,^^32^

Some limitations should be noted.

We focus on the accounts of men diagnosed with mpox and the care they received. We do not attempt to assess whether clinical guidelines for mpox testing/treatment were followed. Rather we provide insights into patient experience to explore how care might be improved. We therefore do not seek to judge the appropriateness of guidelines or individual clinical practice during the outbreak.

Interviews were conducted between 8 and 12 months post diagnosis/recovery, allowing a greater examination of long-term emotional and physical impacts of mpox. However, while experiences were highly distinct and therefore memorable, some had difficulties recalling specific details, especially types of medication provided.

Although we recruited a substantial number of non-UK born GBMSM, we found no differences in their experiences compared to UK born men. Further, our study sample is predominantly White and all participants spoke English to some degree of fluency. This research therefore does not reflect the experiences of those from other ethnic backgrounds and those with limited English language skills who may have had unique experiences navigating care. Further, although we initially planned to include trans women and non-binary people, none were captured by our recruitment strategy, likely because of small numbers of diagnoses in these groups in England.^33^

In order to focus on the voices of those personally impacted by mpox, we used accounts of stakeholders working in the response to triangulate GBMSM perspectives. Conducting a greater number of interviews with a more diverse sample of stakeholders would have provided deeper insights into the service level pressures which shaped patient care.

Because of concerns with potential participant identification we are not able to report time period of diagnosis for all men. We therefore provide these data only when relevant for interpretation and when there is no ethical risk around disclosure.

Finally, our recruitment strategy did not identify individuals who experienced ‘social hospitalisation’ whereby they were admitted in response to broader life circumstances including acute poverty or not being able to isolate from others effectively. This is a significant research gap as these individuals may be especially vulnerable.

In this study, stigma was a central feature of mpox illness for GBMSM and could be exacerbated or lessened depending on the clinical and social support provided. Strengthening cultural competence in working with marginalised communities is critical in improving well-being while addressing (re)emerging infectious diseases. To achieve this, communities should be centrally involved in co-producing, planning and delivering care from the outset of outbreaks.

## Contributors

Study conceptualisation: TCW, AJR, FMB, MP, JS Study oversight: TCW, JS Participant and public involvement: AS Instrument development: TCW, MP, JS, AJR, FMB, CS, RP, AS Recruitment: TCW, JS, HC, KS, KF, JV Data generation: TCW, AG Data analysis: TCW, AG, RP Drafting: TCW, AG, RP, AJR, FMB Critical input: TCW, AG, RP, HC, AJR, CS, AS, ERMP, RV, KS, KF, FMB, JS All authors approved the final manuscript.

## Data sharing statement

All study instruments are provided in supplementary files. Due to their identifying nature, underlying data are not being made available. The corresponding author will consider reasonable requests.

## Declaration of interests

TCW reports grant funding from the Wellcome Trust, NIHR and the European Union Horizon 2020. TCW and CS report honoraria for preparing educational materials from Gilead Sciences. CS reports honoraria for preparing education and presentation materials from ViiV healthcare.
