# Supplementary material for: Experiences of mpox illness and case management among cis and trans gay, bisexual and other men who have sex with men in England: a qualitative study
Source: eClinicalMedicine. 2024 Mar 12;70:102522. doi: 10.1016/j.eclinm.2024.102522 (PMC11056388; doi:10.1016/j.eclinm.2024.102522)
Supplement: Supplementary 1 [file mmc1.docx]

**Understanding the
experiences of illness in
people diagnosed with mpox (monkeypox)**

**We are inviting you to take part in an interview study about mpox (monkeypox)**

- To take part in this study you must first fill in the survey at the end of this form to confirm your eligibility. If you meet the study criteria you will then be contacted by a researcher to arrange an interview.
- We will give you £30 to compensate you for your time if you take part in an interview as part of this research study.
- You are free to decide whether or not to take part in this study. If you choose not to take part, this will not affect the care you get from your own doctors in any way.
- Please take time to read the following information carefully. Discuss it with friends and relatives if you wish. Take time to decide whether or not you wish to take part.
- You can stop taking part in the study at any time, without giving a reason. This can be during the interview or after it is finished.
- Ask us if there is anything that is not clear or if you would like more information.
- Thank you for reading this information. If you decide to take part, you will be given a copy of this information sheet and asked to provide consent electronically.

**Important things that you need to know**

- We want to find out about the experiences of people who have had mpox in the 2022 outbreak
- We are looking to understand more about you as a person, what care and support you received compared to what you needed, and how you felt about having mpox
- This study involves being interviewed - either over the phone or by video calling (e.g. over Zoom) - by an experienced social scientist working in the field of sexual health

| 1 | **Why is this study being done?** |
| --- | --- |

This study is being run in response to the outbreak of mpox in the sexual networks of cis-gender gay, bisexual and other men who have sex with men, trans and non-binary people.

What is this study trying to find out?

We’re trying to understand more about the experiences of people who have had mpox. We want to identify how health and social support can be improved to better meet the needs of people impacted by mpox. We are also interested in how people who have been affected think about mpox generally, and in comparison to other sexually transmitted infections.

| 2 | **Why am I being asked to take part?** |
| --- | --- |

You are being asked to take part in this research because records show you have recently had mpox, or because you identified yourself to researchers as someone who has previously had mpox.

| 3 | **What does taking part involve?** |
| --- | --- |

Can I definitely take part?

Not everyone will be able to take part in this study. We are selecting individuals based on various factors, including the severity of their illness and their migration history.

What will happen to me during the study?

First you will fill in a brief survey providing some information about yourself, including your contact details, so that we can make sure you are eligible. You may then be asked to take part in an interview. This will be over the phone or by video calling using Zoom or Microsoft Teams. During the interview we will ask you questions about your background and sexual history, about how you were diagnosed with mpox and your experiences of care.

Will I get any compensation?

We will compensate you £30 for taking part in this study. This could be through a bank transfer, through PayPal or as a voucher from Amazon.

| 4 | **What are the possible benefits of taking part in this study?** |
| --- | --- |

You may learn more about mpox and sexual health than you did before you participated. You may also find it interesting or helpful to talk about your experiences with our researchers.

| 5 | **What are the possible disadvantages and risks of taking part?** |
| --- | --- |

The risks of taking part in this study are mostly to do with negative thoughts or feelings you might have, particularly if you get upset when talking about issues related to mpox and/or sexual health. This might be especially true if you found your time having mpox to be very distressing. You should consider whether you think the subject matter likely to come up in the interview will be very upsetting for you.

If there are any questions that you find distressing, you don’t need to answer them.

| 6 | **More information about taking part** |
| --- | --- |

Do I have to take part in the study?

No, it is up to you to decide whether or not to take part. If you decide to take part you will be asked to provide electronic consent before the interview starts. You will have the opportunity to discuss this study with the researcher before you consent and can withdraw at any time.

What will happen to information about me collected during the study?

If you agree to take part in an interview the researcher will record the conversation. This recording will then be written up word-for-word by a specialist agency bound by the same confidentiality rules as the researcher. Any identifying names or places that you mention during the interview will be changed to protect your confidentiality. If any quotes are taken from what you say we will not mention your name or attribute them to you in any way. The information will be analysed by the researchers and will be password protected for the duration of the study.

The information we collect about you will be useful in future research. Other researchers, including some who may be working outside England, may ask to use anonymised data collected during this study. If they do, this will be considered very carefully by researchers involved in this study, and independent scientists. We will follow all legal requirements to make sure that all information about you is treated appropriately and ethically, and that other researchers do so too. We will not consider data access requests from outside Europe.

If you do not participate in an interview none of your data will be shared.

The Institute for Global Health will store your data for 10 years, and then transfer it to the secure UCL data archive in line with GDPR legislation. Access will be controlled by the researchers from this study. There is a question about this during the online consent process we will ask you to sign before you begin the study. We would keep this information separate from other information we collect about you.

‘Public interest task’ and ‘scientific and historical research’ are the lawful basis for processing your information under GDPR rules.

You can find more information on how we use and process your data in line with GDPR requirements here: https://www.ucl.ac.uk/legal-services/privacy/ucl-general-privacy-notice-participants-and-researchers-health-and-care-research-studies

You can find out more about how we use your information:

- by asking the researcher named on this sheet
- by emailing the UCL Data Protection & Freedom of Information Officer: data-protection@ucl.ac.uk

Can I stop taking part after I’ve joined the study?

You can stop taking part in this study at any time and without giving a reason. You can do so by contacting the researcher named on this sheet.

If you stop taking part in this study within 3 months of your interview, we can delete any data we may have collected. Beyond this time, we may not be able to do so as it will be in the process of being published in the academic press.

What will happen to the results of this study?

When the study is completed we will publish the results in a medical journal, so that other researchers can see them. You can ask us for a copy of any publication. Your identity and any personal details will be kept confidential. No named information about you will be published in any report of this study.

Who is organising and funding the study?

This study is organised by The Institute for Global Health at UCL. Funding comes from the National Institute of Health and Care Research (NIHR). This research is being conducted in partnership with the UK Health Security Agency.

Who has reviewed this study?

This study has been reviewed and approved by the UKHSA Research Ethics and Governance Group (Ref:522). This review is to protect your safety, rights and well-being.

What if something goes wrong for me?

If you have any concerns about the way you have been approached or treated during the study, please contact Charles Witzel to discuss.

Email: c.witzel@ucl.ac.uk

If you are harmed by taking part in the study, or if you are harmed because of someone’s negligence, then you may be able to take legal action.

| 7 | **Contacts for further information** |
| --- | --- |

If you want further information about this study, please contact Charles Witzel.

Dr Charles Witzel

c.witzel@ucl.ac.uk

Institute for Global Health

University College London

Royal Free Hospital

Rowland Hill Street

London

NW3 3PF

Thank you for taking the time to consider taking part in this study.

| 8 | **Consent to participate** |
| --- | --- |

If you are interested in participating, please click here to continue to the recruitment survey.

| 9 | **Survey** |
| --- | --- |

1. What is your age

[drop down box] (if under 18 direct to *Exit page - ineligible*)

1. Have you lived in England at any point since May 2023?
   - Yes
   - No [*ineligible]*
2. How do you describe your ethnicity?

Asian or Asian British

- - Indian
  - Pakistani
  - Bangladeshi
  - Chinese
  - Any other Asian background

Black, Black British, Caribbean or African

- - Caribbean
  - African
  - Any other Black, Black British, or Caribbean background
  - Mixed or multiple ethnic groups
  - White and Black Caribbean
  - White and Black African
  - White and Asian
  - Any other Mixed or multiple ethnic background

White

- - English, Welsh, Scottish, Northern Irish or British
  - Irish
  - Gypsy or Irish Traveller
  - Roma
  - Any other White background

Other ethnic group

- - Arab
  - Latin American
  - Any other ethnic group

1. What is your gender?
   - Male (including trans male)
   - Female (including trans female)
   - Non-binary
   - Other (write in: ______)
2. Is this the same as the sex you were assigned at birth?
   - Yes
   - No
3. How do you describe your sexual orientation?
   - Gay / homosexual
   - Bisexual
   - Straight
   - Queer
   - In another way
4. [*if cis-gay man*] Have you had sex with men in the past 12-months?
   - Yes
   - no
5. Were you born in the UK?
   - Yes
   - No
6. [*if not born in the UK*] which country were you born in?
   - [country list]
7. Have you tested positive for mpox (monkeypox) since May 2022?
   - Yes
   - No (*Exit page - ineligible*)
8. When were you diagnosed with mpox (monkeypox)?
   - [date box]
9. Did you have to spend time in hospital (overnight or longer) because you were ill with mpox (monkeypox)?
   - Yes
   - No
10. Which of the following languages would you prefer to be interviewed in?
    - English
    - French
    - Spanish
11. Are you interested in taking part in this study?
    - Yes
    - No (*Exit page-ineligible*)
12. Do you consent to us processing the information you have provided?
    - Yes (*Exit page – eligible)*
    - No (*Exit page - ineligible*)
13. What is your name? (mandatory)
    - Write-in
14. What is your email address? (mandatory)
    - Write-in
    - What is your phone number? (optional)

| 10 | **Exit page - ineligible** |
| --- | --- |

Thank you for taking the time to fill in the survey and for your interest in this research.

The answers you have provided us with indicate you are either not eligible for this study or you do not wish to participate. All data you have provided has been securely deleted and we will not contact you.

If you are interested in learning more about mpox, please visit this NHS website which provides more information. [Hyperlink: https://www.nhs.uk/conditions/mpox/]

If you require support please contact Terrence Higgins Trust Direct [Hyperlink: <https://www.tht.org.uk/our-services/support-services/tht-direct-helpline>] or by calling 0808 802 1221 10am-6pm Monday-Friday.

| 10 | **Exit page - eligible** |
| --- | --- |

Thank you for taking the time to fill in the survey and for your interest in this research. We will now assess your eligibility for the study. If you meet the current inclusion criteria we will invite you to an interview about your experiences.

If you have any questions, please contact the study principle investigator Charles Witzel at [c.witzel@ucl.ac.uk](mailto:c.witzel@ucl.ac.uk).

If you are interested in learning more about mpox, please visit this NHS website which provides more information. [Hyperlink https://www.nhs.uk/conditions/mpox/]
