# Supplementary material for: Experiences of mpox illness and case management among cis and trans gay, bisexual and other men who have sex with men in England: a qualitative study
Source: eClinicalMedicine. 2024 Mar 12;70:102522. doi: 10.1016/j.eclinm.2024.102522 (PMC11056388; doi:10.1016/j.eclinm.2024.102522)
Supplement: Supplementary 2 [file mmc2.docx]

**GBMSM with confirmed mpox topic guide**

**INTRODUCTION**

- interviewer to introduce self
- go through information sheet / review consent form
- go through verbal consent, recording, translation and transcription.
- Do you have any questions?
- would you like a copy of the findings?
- Explain that mpox / mpox will be used interchangeably throughout the interview

**EXPERIENCE OF MPOX ILLNESS, DIAGNOSIS AND CARE**

1. In this section we’re going to talk about your experiences of having mpox. Can you tell me a bit about how you found out you had mpox?
   - What were the reasons for testing (symptoms, routine testing, partner notification)?
     - (*If not answered*) what kinds of symptoms did you have? At any point did you have a fever? How about lesions?
     - (*if* relevant) Can you tell me about what having mpox lesions was like for you? How did it make you feel?
   - Describe for me the experience of going to the clinic to get tested.
     - (*if not answered*) When and where were you tested and diagnosed?
     - Did you feel like your testing and diagnosis with mpox was as quick or efficient as you wanted or needed it to be?
     - (*if relevant*) What would have improved the experience for you?
     - What kind of PPE were the clinical staff wearing and what were your feelings about it?
     - Did the health care workers answer your questions? Did you have any questions that weren’t answered?
     - (*Probe*): What did you find most helpful? What was least helpful?

- When you were diagnosed with mpox, were there any details about your sex life that you did not feel comfortable sharing with the doctor or nurse?
  - - Can you tell me what you felt uncomfortable sharing?

1. We’re now going to talk a bit more about your experiences of treatment.
   - First off, I’m wondering what kind of treatment you received, if any?
     - Is this what you wanted?
     - What other things might have been helpful to you in your situation?
     - Were you offered vaccination to reduce the symptoms?
   - (*if hospitalised*) I see from the answers you provided before the interview that you were hospitalised. Can you tell me a bit about what that experience was like for you?
     - What were the reasons for your hospitalisation?
     - Did you receive the care that you felt you needed while in hospital?
     - (*if relevant*) what other things might have helped you at that time?
   - Can you tell me a bit about your experience of isolating while you were sick?
     - What kinds of challenges did you face while you were isolating?
     - (*if relevant*) Was it difficult to stay away from housemates / partner(s) / family?
     - Did isolating have any impact on your employment or education? Can you describe it for me?
     - What kind of support, if any, did you receive while isolating? Is this what you needed?
2. After a mpox diagnosis, some people are helped by the clinic or by public health teams to contact their close contacts, including their sexual partners.
   - What was your experience of getting in touch with partners like?
     - What was the process for contacting these people? Who did it?
     - Did it happen through face-to-face, phone, or text?
     - How did the process make you feel? Is there support that could or should have been offered but that wasn’t?
3. We’re now going to discuss your feelings about having monkey pox.
   - Can you tell me a bit about what your immediate reaction was when you were diagnosed?
     - (*if relevant*) how did these feelings compare to previous diagnoses of STIs that you’ve had, if at all?
     - Did you feel like you were able to discuss your diagnosis with people close to you?
     - Can you tell me a bit about how you made decisions around who to tell and what information to share?
     - If disclosed, how did other people react when you told them you had mpox?
     - What impact, if any, did how mpox is portrayed in the media have on how you felt?
   - What support, if any, did you get from your friends or family when you had mpox?
     - What additional support would have been helpful?
4. Some people find that being diagnosed with mpox has an impact on how they feel about sex.
   - Could you tell me what changes, if any, being diagnosed with mpox has made to the sex you have had since?
     - (*if relevant*) When did you make these changes? How long have they lasted for?
     - What kind of advice have you received from healthcare workers about sexual activity?
     - (*if* relevant) How have you felt about that advice?
     - (*if not* answered) Do you feel that advise is realistic or feasible for you to follow?
5. We’re coming to the end of the interview now and I have some final questions about mpox knowledge before and after diagnosis.
   - What did you know about mpox before you were diagnosed?
     - (*if relevant*) Had you heard of it?
     - Did you know much about transmission, severity, symptoms, treatment, prevention?
     - Where had you learned about mpox from? (NHS material, friends, media)
     - What were your thoughts about mpox? (Feelings, stigmatized, severity, relate this to previous question on feelings upon diagnosis)

- Was it something that you were concerned about getting personally? (What if anything did you do to avoid it?)
- Since your mpox diagnosis, what have you learned about it? Where did you learn this from? What did you find most useful? Why? Have your feelings about it changed?

**PERSONAL BACKGROUND**

1. Finally, I’m going to ask you some background questions about yourself. This helps us to understand a bit more about your life and experiences. There are no wrong answers to any of these questions, we just want to understand more about you.
   - Where were you born? What is your first language?
   - How would you best describe your ethnicity?
   - Where do you currently live?
   - Had you been in contact with sexual health services prior to your mpox diagnosis?
     - How often did you use these services?
     - Besides mpox, have you had any other sexually transmitted infections in your lifetime?
     - Do you know your HIV status? (prompt to share)
       - (*if HIV positive)* Which year where you diagnosed?
       - (*if HIV positive)* Is there anything you would like to share about your HIV diagnosis?
       - (*if HIV negative*) Are you taking PrEP? Have y+ou done so in the past?
   - Do you use any drugs recreationally? Which kinds/methods?
     - (*if relevant*) when and how do you use drugs? E.g. sexual settings, with friends, both?
     - (*if methamphetamine*) Can you tell me how you take crystal meth? Do you slam or have you slammed in the past?
   - Can you tell me a bit about your attitudes towards condoms?
     - (*if* relevant) About what proportion of the time do you think you use condoms?
   - Have you ever had sex in exchange of money, goods, shelter or food?
     - (*if yes*): Where do you meet clients?
     - *(Probe if yes*): How do you decide whether to use condoms and when do you not? And what about your partners?
     - (*Probe if yes*): Do you feel safe?

**Stakeholder topic guide**

**INTRODUCTION**

- interviewer to introduce self
- go through information sheet / review consent procedure
- Send consent link: https://redcap.idhs.ucl.ac.uk/surveys/?s=M7LCPA79PRR9KWWL
- Ask participant to fill out consent on redcap (provide link)
- go through electronic consent, recording, translation and transcription.
- Do you have any questions?
- would you like a copy of the findings?
- Obtain consent

**PERSONAL BACKGROUND**

1. First off, I’m going to ask you some background questions about your role and organisation. This helps us contextualise some of your answers when we analyse our data.
   - Where do you currently work?
   - Can you describe your role for me?
     - How, if at all, has this changed during the mpox outbreak?
     - (*if not* answered) Have you had a direct role in the response?

**OUTBREAK PREPARDNESS**

1. In this section we’re going to talk about the mpox response.
   - Overall, to what extent do you feel the sector was prepared for an outbreak of a pathogen primarily impacting GBMSM, trans and non-binary people?
     - What, if anything, would have improved your ability to respond to the outbreak?
     - How, if at all, have you worked with the community in responding the MPOX?
     - (*if relevant*) Is this different to your usual practice?
   - Can you tell me what your organisation’s main approach to dealing with the mpox outbreak has been?
     - What types of activities have you started doing that you weren’t doing before?
     - Can you tell me what the main barriers have been in responding to the outbreak?
     - (*if* relevant) How about facilitators?
     - (*if* relevant) Is there anything else you would have liked to put in place but haven’t been able to?
     - (*if relevant*) What, if any, resources or materials would have been helpful in responding to the outbreak?

**MPOX: PATIENT’S NEEDS FOR SUPPORT**

1. We’re now going to discuss the needs of people who have had mpox, and how to support them and address those needs. This includes support for patients across a range of areas, from support related to their physical and mental health and wellbeing, to social and financial support.
   - In your experience responding to this outbreak, what are the needs of people diagnosed with mpox that you have identified?
     - (*if relevant*) What about needs related to their physical health and wellbeing?
     - (*if relevant*) What about needs related to their mental and emotional health and wellbeing?
     - (*if relevant*) What about needs related to their social lives, including social support?
     - (*if relevant*) What about needs related to their financial wellbeing?
   - Can you tell me about how organisation/clinic provides support to individuals who have (or potentially have) mpox?
     - Can you tell me about any unaddressed health and social support needs that you can identify?
     - (*if relevant*) What are the primary barriers and facilitators to responding to these?

**MPOX AND STIGMA**

1. We’re now going to talk a bit about the relationship between the mpox outbreak and stigma.
   - Could you tell me about how, if at all, you think mpox is linked with stigma?
     - (*if relevant*) how do you think stigma associated with mpox intersects with other types of stigma?
     - (*if relevant*) How about in relation to HIV, STIs, sex between men etc?
     - (*if relevant*) Can you tell me how, if at all, the media has shaped or influenced mpox stigma?
     - (*if relevant*) what types of activities or interventions do you think are important or helpful in reducing stigma?

- How is stigma towards mpox is similar to stigma towards HIV? How is it different?
  - To what extent do you think the mpox response may have been hindered by stigma?
  - What, if anything, would you want to be done to address stigma?

**WRAP-UP**

1. That’s all the questions I have for you today.
   - Is there anything you’d like to share? Anything you think we’ve missed?
   - Thank you so much for your time, it is really appreciated.
