# Supplementary material for: Experiences of mpox illness and case management among cis and trans gay, bisexual and other men who have sex with men in England: a qualitative study
Source: eClinicalMedicine. 2024 Mar 12;70:102522. doi: 10.1016/j.eclinm.2024.102522 (PMC11056388; doi:10.1016/j.eclinm.2024.102522)
Supplement: Supplementary 3 [file mmc3.docx]

| **Meta-theme: Social and emotional impacts of mpox** | | |
| --- | --- | --- |
| **Theme** | **Sub-theme** | **Explanation** |
| From one pandemic to the next | Comparisons with HIV | - Revival of the HIV diagnosis announcement (individual level, denial) - Revival of the beginning of the HIV epidemic (collective level, ‘community anxiety’) - New ‘gay disease’ - Government failings, ineffective because the epidemic was concentrated among gay people - Gay people and doctors were discovering a new disease together, complicity |
|  | Comparisons with COVID-19 | - Widespread panic at the very beginning of the epidemic - Isolation (mpox) / lockdown (Covid-19); ppsychological impact of isolating |
| Emotional reactions: fears and resilience | Initial reactions | - Disbelief, upset |
|  | Fears | - Unknown disease course, potential worsening - stigmatising lesions, pain and potential stigmatising scars - Fear was increased by friends’ stories and alarmist information given in the media - No specific treatment was available (unlike other STIs, mpox is a STI “like no other”) |
|  | Severity | - Comparisons to other STIs |
|  | Resilience | - - Resilience from prior life experience key in coping with diagnosis/illness   - Older GBMSM with diagnosed HIV and more experience of managing stigmatising illness coped better     - Younger GBMSM without diagnosed HIV had more challenges. |
| Intersecting, multiple stigmas | Internalised stigma | - Feeling ‘dirty’ (stigmatising lesions, anal pain, direct link with sexual behaviours) - Finding the response after disclosure to not be as judgemental as anticipated – self-stigma and internalised homophobia a substantial problem. - Change in sexual practices after illness… as a global questioning of sexuality |
|  | Anticipated stigma | - Concerns about judgement after disclosing diagnosis (at work, with friends, with sex partners and/or main partner). |
|  | Felt or enacted stigma | - Negative reactions from others - Impact of social and news media |
| Relationships, abstinence and reinitiating sexual behaviour | Relationships | - Impacts on primary relationships - Relationship termination (or not) |
|  | Sex | - Impacts on how people felt about sex - Feelings of loss of control, shame and internalised stigma - Guidelines around condom use following recovery |
| **Meta-theme 2: Clinical pathways, case management and recovery** | | |
| **Theme** | **Sub-theme** | **Explanation** |
| Initial illness, testing and diagnosis | First symptoms | - Which symptoms came first - Pain, lesions, fever etc |
|  | Testing process | - Journey through clinics and testing - Denied testing - Incidental diagnosis during routine testing - PPE and reactions to it |
| Treatment, contact tracing and public health | Clinics | - Service provision and pain management - Experiences of contact tracing - Vaccination following recovery - Answering questions and providing information |
|  | Health protection | - Service responsiveness - Contact tracing - Vaccinating contacts - Preferences for delivery in future |
| Isolation and recovery | Course of illness | - How bad it got, symptoms etc |
|  | Discussions with others | - Family - Friends - Managing disclosures |
|  | Isolation | - Emotional and practical challenges - Distance from others: how to maintain - Strategies |
|  | Employers | - Supportive vs not - Inappropriate disclosures |
| Longer-term impacts |  | - Longer term impacts - Access services - Mental health |
| **Miscellaneous** |  |  |
| Demographics |  | - Anything about demographic background |
| Condom use |  | - Data around condom use |
| Information sources |  |  |
