# Supplementary material for: Experiences of mpox illness and case management among cis and trans gay, bisexual and other men who have sex with men in England: a qualitative study
Source: eClinicalMedicine. 2024 Mar 12;70:102522. doi: 10.1016/j.eclinm.2024.102522 (PMC11056388; doi:10.1016/j.eclinm.2024.102522)
Supplement: Supplementary 4 [file mmc4.docx]

## Appendix

The NIHR HPRU in BBSTI Steering Committee consists of: Professor Caroline Sabin (HPRU Director), Dr John Saunders (UKHSA Lead), Professor Catherine Mercer, Dr Hamish Mohammed, Professor Greta Rait, Dr Ruth Simmons, Professor William Rosenberg, Dr Tamyo Mbisa, Professor Rosalind Raine, Dr Sema Mandal, Dr Rosamund Yu, Dr Samreen Ijaz, Dr Fabiana Lorencatto, Dr Rachel Hunter, Dr Kirsty Foster and Dr Mamoona Tahir.
